# The Oncopig Cancer Model: An Innovative Large Animal Translational Oncology Platform

**DOI:** 10.3389/fonc.2017.00190

**Published:** 2017-08-23

**Authors:** Kyle M. Schachtschneider, Regina M. Schwind, Jordan Newson, Nickolas Kinachtchouk, Mark Rizko, Nasya Mendoza-Elias, Paul Grippo, Daniel R. Principe, Alex Park, Nana H. Overgaard, Gregers Jungersen, Kelly D. Garcia, Ajay V. Maker, Laurie A. Rund, Howard Ozer, Ron C. Gaba, Lawrence B. Schook

**Affiliations:** ^1^Department of Radiology, University of Illinois at Chicago, Chicago, IL, United States; ^2^Albion College, Albion, MI, United States; ^3^College of Medicine, University of Illinois at Chicago, Chicago, IL, United States; ^4^Department of Medicine, University of Illinois at Chicago, Chicago, IL, United States; ^5^Division of Immunology and Vaccinology, National Veterinary Institute, Technical University of Denmark, Kongens Lyngby, Denmark; ^6^Biologic Resources Laboratory, University of Illinois at Chicago, Chicago, IL, United States; ^7^Department of Surgical Oncology, University of Illinois at Chicago, Chicago, IL, United States; ^8^Department of Animal Sciences, University of Illinois, Urbana, IL, United States

**Keywords:** cancer models, pigs, oncopig, clinical needs, oncology, translational medicine

## Abstract

Despite an improved understanding of cancer molecular biology, immune landscapes, and advancements in cytotoxic, biologic, and immunologic anti-cancer therapeutics, cancer remains a leading cause of death worldwide. More than 8.2 million deaths were attributed to cancer in 2012, and it is anticipated that cancer incidence will continue to rise, with 19.3 million cases expected by 2025. The development and investigation of new diagnostic modalities and innovative therapeutic tools is critical for reducing the global cancer burden. Toward this end, transitional animal models serve a crucial role in bridging the gap between fundamental diagnostic and therapeutic discoveries and human clinical trials. Such animal models offer insights into all aspects of the basic science-clinical translational cancer research continuum (screening, detection, oncogenesis, tumor biology, immunogenicity, therapeutics, and outcomes). To date, however, cancer research progress has been markedly hampered by lack of a genotypically, anatomically, and physiologically relevant large animal model. Without progressive cancer models, discoveries are hindered and cures are improbable. Herein, we describe a transgenic porcine model—the Oncopig Cancer Model (OCM)—as a next-generation large animal platform for the study of hematologic and solid tumor oncology. With mutations in key tumor suppressor and oncogenes, *TP53^R167H^* and *KRAS^G12D^*, the OCM recapitulates transcriptional hallmarks of human disease while also exhibiting clinically relevant histologic and genotypic tumor phenotypes. Moreover, as obesity rates increase across the global population, cancer patients commonly present clinically with multiple comorbid conditions. Due to the effects of these comorbidities on patient management, therapeutic strategies, and clinical outcomes, an ideal animal model should develop cancer on the background of representative comorbid conditions (tumor macro- and microenvironments). As observed in clinical practice, liver cirrhosis frequently precedes development of primary liver cancer or hepatocellular carcinoma. The OCM has the capacity to develop tumors in combination with such relevant comorbidities. Furthermore, studies on the tumor microenvironment demonstrate similarities between OCM and human cancer genomic landscapes. This review highlights the potential of this and other large animal platforms as transitional models to bridge the gap between basic research and clinical practice.

## Introduction

Cancer is a global epidemic causing more than 8 million annual deaths worldwide. The more than 13 million new cancer diagnoses made each year carry an economic burden of $290B. Cancer is expected to be the second leading cause of death in the United States in 2017. The American Cancer Society (ACS) estimates approximately 1,688,780 new cancer diagnoses will be made and 600,920 Americans will die from cancer. The Agency for Healthcare Research and Quality (AHRQ) estimates that direct medical costs of cancer in the United States in 2014 exceeded $87B. Many of these diagnoses, deaths, and costs could be avoided by shortening the gap between pre-clinical research and regulatory approval for safe and effective therapies. Large animal models that closely recapitulate human cancer and comorbid diseases represent a critical tool in the global cancer-fighting toolbox.

Cancers are deadliest when diagnosed at late stages, a problem that is caused by lack of early detection tests. Cancers of the colon, esophagus, liver and intrahepatic bile ducts, lung and bronchus, non-Hodgkin lymphoma, oral cavity, ovary, pancreas, and uterine cervix are diagnosed at regional or distant stages in more than 50% of cases, resulting in poor survival for many patients. The journey for advancing cancer diagnostics and therapeutics is both lengthy as well as expensive. On average, it takes approximately 8 years, at a cost of $1.2B, per approved antineoplastic agent to complete the required series of clinical trials leading to regulatory approval. This timeline and cost does not include pre-clinical development and testing, thereby establishing that the total time from development to approval exceeds a decade, per agent. Approximately 10% of drugs that begin pre-clinical testing advance into human testing. Roughly 75% of research and development costs are attributed to failures throughout the drug discovery process, leading to the perception that drug discovery and development is one of the most precarious financial undertakings in science and biomedical research. However, with better models that recapitulate human conditions, fewer of these failures may be observed in human clinical trial participants. Better models offer the promise of shortening the timeline for pre-clinical and clinical trials, as well as substantially reducing the cost. Perhaps the most outstanding opportunity is to observe such failures in a large animal model, saving years in patient accrual to human clinical trials and millions of dollars to conduct such trials.

The concerns of bringing new drugs to market are recognized by pharmaceutical companies, physicians, researchers, and perhaps most importantly, patients. In recent years, the U.S. Food and Drug Administration (FDA) has worked diligently to decrease the timelines for approval while increasing the number of new drug approvals. Since 2015, 39 new indications on more than 20 newly approved drugs have come into effect. In considering a new agent for approval, the FDA does not calculate the cost-effectiveness of the agent under review. Many newly approved therapies carry a substantial cost exceeding $60,000–$120,000 annually, high price tags for marginal clinical benefits. Unfortunately, due to the lengthy timeline and expensive costs of developing drugs, many costs are passed on to insurance companies and patients. Utilization of large animal models that best mimic human diseases improves the potential for those agents, which reach human trials to have a better chance for success, eventually leading to fewer development costs for the market to bear.

With specific regard to cancer therapy, costs are compounded as patients eventually fail first-line therapy, thereby moving onto second-, third-, and fourth-line therapies, and so on, until treatment options are exhausted. As patients and physicians desperately hope for cures, off-label treatments are frequently employed on a case-by-case basis. The army of available antineoplastic agents, which has grown substantially following the sequence of the human genome, further increases costs while confounding guideline-driven treatment. Pre-clinical or co-clinical large animal models play an important role in the process of new drug trials and approvals. As the biomedical research community works to make the promise of precision medicine a reality, better animal models are more critical now than ever before.

Animal models, and specifically mouse models, have played a major role in our understanding of the genetic basis of cancer and the role of specific genes and gene mutations in the development and progression of cancer. However, gaining a complete understanding of cancer, which reflects an astonishing number of variant diseases, and translating this knowledge to more efficacious treatments and cures have been elusive. In a clinical landscape that is already challenging, the promise of precision cancer medicine serves to further complicate cancer therapeutics. Precision medicine, simply defined as the right treatment for the right patient, at the right time, demands highly relevant translational models to recapitulate human disease. As clinical practice is being driven more and more by molecular pathology, the treatment landscape becomes unique for each individual cancer patient, rather than cohorts of patients treated as one. This review highlights the advantages and disadvantages of currently available small and large animal cancer models and introduces the Oncopig Cancer Model (OCM) as a qualified alternative to currently available cancer models applicable to a wide variety of cancer types.

## Current Small Animal Models

Advances in cancer care are dependent upon the use of pre-clinical *in vivo* model systems to test safety and efficacy. In general, an ideal animal model for biomedical research should: (1) mimic the human disease on a molecular basis, (2) derive from a relevant cell line that lends itself to *in vitro* study, (3) be reliable and predictable, (4) manifest survival differences, (5) allow for accurate treatment assessment, (6) be readily imaged, and (7) occur in similar background settings as the human disease (1). A variety of *in vivo* systems have been used to study cancer biology including the development of genetically modified rodents, immunodeficient mouse models engrafted with human tumors, and the use of carcinogens and radiation to induce tumors (2–5). However, due to vast differences between humans and rodents such as mice, the ability to model complex diseases such as cancer and translate results to clinical practice is quite limited (6). This section focuses on the benefits and drawbacks of currently used small animal cancer models.

### Mouse Models

Murine models offer several advantages such as the availability of a wealth of genetic information, reduced genetic variation, short generation intervals, high fecundity, and ease of maintenance and handling at a more affordable cost. There are also vast numbers of commercially available mouse lines with know genetics, making them highly suitable to model a wide variety of human diseases. While murine models represent the most commonly used small animal cancer models, there are several drawbacks associated with their use. Humans are 3,000 times larger than mice, live 30–50 times longer and, therefore, undergo about 105 more cell divisions in a lifetime (7). Without genetic modification, mice develop cancers of mainly mesenchymal origin, such as sarcomas and lymphomas, whereas humans have a bias toward the development of epithelial cancers (carcinomas) with age (7). The small size and short lifespan of mice, while advantageous for reducing study times and housing needs, means that loss of certain tumor suppressor genes is insufficient to result in the development of cancer in a highly penetrant manner, particularly when such mutations are heterozygous. Accordingly, investigators have used the Cre-Lox system to homozygously inactivate tumor suppressors in a tissue or cell type-specific manner. While this is often sufficient to drive tumor formation, such a situation does not mimic the cancer disease progression for patients in which rare loss of heterozygosity (LOH), a genetic condition in which one copy of a heterozygous genomic region (i.e., gene or genetic locus and portion of chromosome) is lost due to a mutational event occurs. LOH is a common phenomenon in human cancer, which can result in the loss of tumor suppressor gene functions through elimination of the allele encoding the functional copy of a gene in a subset of cells in the body, often leading to the development of a tumor or the progression of an existing tumor. Because mouse chromosomes are telocentric, LOH often occurs in murine models by loss of the entire chromosome carrying the wild-type tumor suppressor gene allele in cells heterozygous for a tumor suppressor gene mutation (8). However, in human tumors, LOH usually occurs via sub-chromosomal deletions covering the wild-type tumor suppressor gene locus (9, 10).

On a cellular level, murine cells have lower thresholds for genetic and/or epigenetic changes that lead to transformation in culture, which further demonstrates fundamental differences in the mechanistic properties of cancer development between mice and humans (11). Arguably, the most profound difference between mouse models and humans is the essentially 100% homozygosity of every locus in inbred mouse lines, which makes extrapolation back to human populations challenging (12). Mouse cells are immortalized much more readily than human cells (7). It has also been suggested that mouse cells respond to oncogenic *Ras* expression differently than human cells; *RAS* oncogenes require Ras-like (Ral) signaling in human cells, whereas the requirement for this signaling pathway is much reduced in *Ras* oncogene transformation of mouse cells (13). Laboratory mouse strains have very long telomeres and express *Tert*, in contrast to human cells (11, 14). Moreover, mice do not develop the same forms of genetic instability that human cells do during tumorigenesis, perhaps due to their shorter lifespan that could restrict the number of sequential mutations that accumulate in human tumors (14).

Organ systems also vary between mice and humans such that certain types of cancer cannot be accurately modeled. For example, anatomical and physiologic variances between the mouse and human pancreas make modeling pancreatic cancers in mice difficult. The human pancreas is a retroperitoneal and segmented organ divided into a distinct head, body, and tail (15). In contrast, the mouse pancreas is diffuse, dendritic, and poorly lobulated (16). While the vascular supply between mice and humans are largely homologous, there are also substantial differences in several of the functional cell types between the two species. In humans, the exocrine pancreatic acini are organized into lobules that secrete to a small, intercalated duct. These then drain to larger, interlobular ducts, which then join to form the main pancreatic duct. This then joins the bile duct and empties to the duodenum. The mouse pancreas has a large interlobular duct that drains the three respective lobes. The splenic and gastric ducts then merge with the common bile duct and empty more proximally to the duodenum (15). The endocrine component of the pancreas also differs. While humans have 1,000–3,000 times more endocrine islets than mice, humans have a larger proportion of glucagon producing α-cells than mice, who have a larger relative percentage of insulin producing β-cells. Human islets are also rich with both parasympathetic and sympathetic innervation and uniformly distributed, while mice have comparatively sparse autonomic innervation and random islet distribution (15). However, despite these differences, the Pdx-Cre x LSL-*Kras^G12D^- Trp53^R172H^* (KPC) mouse has been the benchmark for pancreatic cancer research for the better part of a decade (17). By targeting expression of *Kras^G12D^* and *Trp53^R172H^* mutations to the exocrine pancreas via the Pdx1 promoter, this model produces reliable and clinically relevant cancer histotypes.

Fundamental differences in how tumorigenesis occurs in mice and humans also exist. For example, humans carrying one mutant and one wild-type allele for the tumor suppressor gene *APC* develop polyps in the large intestine that progressively leads to invasive carcinoma. In contrast, mice with the same heterozygous state for *Apc* develop polyps in the small intestine that rarely show disease progression (18). Such differences in cancer development are due to inherent biological differences between man and mice and are not limited to intestinal polyps but are observed in many mouse models of cancer. This is well illustrated by variations in tumor spectrum when certain tumor suppressor genes known to cause specific cancers in humans are knocked out in mice.

The body size limitation of mice makes the development of novel imaging modalities and surgical techniques nearly impossible yet these are key techniques needed to diagnose and treat a wide variety of tumor types in patients. Moreover, the rate of metabolism is substantially higher in mice compared to humans (7). These differences mean that the pathways by which tumor progression occurs can vary dramatically when comparing mouse models to human cancer. As a consequence, the tumors that develop in a mouse model may respond differently to therapy. For these genetic and physiological reasons, including vast differences in drug metabolism and xenobiotic receptors, rodents also poorly model toxicity, sensitivity, and efficacy when used in pre-clinical drug studies (19). The ability to establish toxicity and drug sensitivity pre-clinically in animal models is immensely important because less than 8% of cancer drugs translate successfully in Phase I clinical trials from animal models (20). While mice have provided numerous insights into the biology of cancer, their historical limitations emphasize the need to develop new models for cancer translational research.

In addition to genetic-based cancer models, induction of tumorigenesis via administration of carcinogenic agents is utilized to study cancer in small animal models. However, a major disadvantage of this method is the time from administration of the carcinogenic agent to tumor formation, which can range from 30 to 50 weeks (21). Another route of establishing *in vivo* tumors is xenograft of tumor cell lines into mice. Although this mechanism is temporally practical, the ensuing pathogenesis is not always representative of human disease (21, 22).

### Rat Models

Rats represent another rodent commonly utilized as pre-clinical cancer models. In addition to some of the abovementioned advantages of murine models, rats have the added benefit of larger size, rendering them more amenable to interventions such as surgery and radiological imaging (23). Rats are commonly used to model colon and bone cancers, largely by exposure to chemical carcinogens (23, 24). In addition, surgical manipulations have been utilized to develop rat models of metaplastic reflux-induced esophageal cancer (25). Recent genome-wide association studies in rats have also identified correlations between rat and human genetic markers of cancer risk (26). However, these models are often limited in their ability to recapitulate human cancer pathophysiology. For example, transgenic and xenograft-induced rat breast cancer models exhibit spontaneous necrosis and failure to metastasize (27, 28). In addition, engrafted rat pancreatic neuroendocrine tumors (PNET) exhibit increased tumor growth following treatment with an mTOR inhibitor, a response that contrasts the results of mTOR inhibitor clinical trials (29).

### Zebrafish Models

Zebrafish are one of the few non-mammalian species that have been extensively utilized as cancer models. As a potential model organism, zebrafish exhibit several advantages. The short lifespan and high reproductive capacity of zebrafish render them amenable to high-throughput screening for genetic mutations (30). In addition, the zebrafish genome shares high homology with humans (31), allowing the use of zebrafish tumorigenic mutations to gain insights into human tumorigenesis. The use of gene-editing techniques including Clustered Regularly Interspaced Short Palindromic Repeats (CRISPR) has facilitated mutagenesis of numerous gene loci in this highly reproductive species (32). Zebrafish cell lines also represent valuable *in vitro* models, including models of broad spectrum leukemia using mutant *c-Myc* transgenic zebrafish (33) and malignant melanoma using *BRAF* mutant zebrafish (34). Nevertheless, zebrafish cancer models are not without limitations. Zebrafish exhibit great diversity both across and within strains that results in high levels of individual-specific variation (31). In addition, genomic comparisons between human melanoma patients and zebrafish models have identified reduced mutational burden in zebrafish tumor cells, suggesting significant differences in genomic stability between humans and zebrafish (35). Moreover, attempts to model certain cancers including acute myeloblastic leukemia and pancreatic carcinoma have either failed to develop or exhibit limited metastatic capacity (35). Therefore, zebrafish cancer models exhibit limitations that prevent their use as consistent models of the wide variety of human cancer phenotypes.

### Small Animal Hepatocellular Carcinoma (HCC) Models

In addition to small animal models that are utilized to model many different human cancers, there are animal models whose ability to model human disease is limited to one cancer or cancer subtype. For example, the rabbit VX2 model is one of the most commonly utilized small animal HCC models. In this model, virally infected VX2 carcinoma cell cultures are injected into rabbits resulting in tumor formation in the rabbit liver (36). However, these tumors have unknown biology, varying tumor kinetics, and unknown genome organization (36), highlighting the limitations of this model as a relevant human HCC model. Another drawback of this model is spontaneous tumor necrosis, which confounds the evaluation of treatment response after pharmacological or interventional treatment. This represents a significant drawback for this model, given its use by interventional radiologists for novel locoregional therapy testing. Another commonly used HCC mode is the woodchuck model, which produces HCC tumors in response to woodchuck hepatitis virus (WHV) infection. WHV infection shares many disease characteristics with the human hepatitis B virus (HBV), which causes liver cirrhosis and leads to HCC development in humans. Similarities between WHV and HBV are seen in the morphology of the virus, its life cycle, and the resulting development of HCC after 2–4 years of infection (37). This model has been used to develop radiofrequency ablation of primary HCC tumors in pre-clinical trials (38); however, several limitations exist, including differential behavior (woodchuck’s hibernate for a period of 4–6 months) and variable diet and WHV infection period when using wild specimens (39).

## Current Large Animal Models

Large animal models of cancer comprise a smaller portion of cancer models than small animal cancer models. While small animal models offer several advantages such as the availability of a wealth of genetic information, reduced genetic variation, short generation intervals, high fecundity, and ease of maintenance and handling at a more affordable cost, they do not provide the anatomical scale required to develop interventional treatments. Large animal models such as pigs offer a more anatomically similar organism to develop these interventional treatment (40–42) and offer cancer cell biology more analogous to human cancer cell biology (43, 44). This section focuses on the benefits and drawbacks of currently used and up and coming large animal cancer models.

### Canine Cancer Models

Client owned dogs provide a unique opportunity to study spontaneously developing tumors in a context that is beneficial for both pets and people. In order to utilize client owned dogs to help researchers better understand tumor biology and facilitate translation of novel human cancer treatments to clinical settings, the National Cancer Institute’s Center for Cancer Research started the Comparative Oncology Program (COP) in 2003.^1^ Use of these animals as comparative cancer models is beneficial due to their many biological similarities with humans along with the large genetic diversity observed within the canine population. Tumors commonly presenting in dogs include osteosarcoma, soft tissue sarcomas (STS), lung carcinoma, oral melanoma, mammary carcinoma, oral squamous cell carcinoma, nasal tumors, and malignant non-Hodgkin’s lymphoma—likely the best cancer model provided by canines because it has considerable analogy to the human variant. Canine cancer models are unique because they spontaneously present with tumors with several characteristics similar to those observed in humans (i.e., osteosarcoma in large breed dogs) (45). Indeed, cancer occurs naturally in dogs with rates reported to range between 5 and 33% (46, 47). It is estimated that 45% of dogs 10 and older die of cancer (45), which is comparable to the estimated 60% of humans who are diagnosed with cancer of the age of 65 years (48). This natural occurrence and history of cancer permits the rapid study of DNA damage and epigenetic alterations that accumulate over time to result in tumor formation, especially given the high homology observed between the canine and human genome (49). Because of these advantages, researchers have been able to utilize canine cancer models to identify relevant genetic alterations and drivers of cancer similar to those observed in human cancers. Additionally, canine subjects bypass the phases of clinical trial testing, accelerating the pace of drug development (50). Many drugs have undergone pre-clinical trials using canine cancer models including Resiniferatoxin, a drug that acts as an agonist for pain caused by bone cancer, due to the canine’s highly noticeable response of self-mutilation of areas in pain (51).

There are several disadvantages with using dogs to model human cancers. Canine cancer models tend to consist of lymphoid and sarcoma tumor types as opposed to carcinomas. Cancer drug development studies conducted in canines are also not always translatable to humans, as dogs have varying drug sensitivity compared to humans (52). Another contention surrounding the use of canine cancer models in translational research is the issue of outbred versus inbred models; because modern dog breeds are a product of line inbreeding, their ability to provide a relevant model of diverse and heterogeneous human cancers is questionable (47). Finally, accrual of client dogs to clinical trials—as with human patients—presents a barrier to the timeliness of study conduct.

### Non-Human Primate Models

To date, published reviews or studies on cancer in non-human primates are relatively scarce and limited to single case reports and small case studies. However, there has been a steady increase in the number of reviews published on cancer in non-human primates (53–56). These reviews likely represent an increase in the recognition of cancer in non-human primates, but they also likely represent an increase in the longevity of non-human primates maintained in research facilities attributed to factors such as improved health care and nutrition and improvements in record keeping, including breeding history, genetic background, and clinical course of disease.

Potentially, non-human primates offer advantages for studying cancer because of their anatomical, physiological, and genetic similarities with humans, being the only bipedal mammalian animal model for research and having 1:1 homology with the majority of human protein-coding genes (57). It is difficult to determine the concordance of toxicities identified in non-human primates relative to humans and other species because of a lack of clinical data. It is tempting to assume that in response to drug delivery, non-human primates will have pharmacological or physiological responses most similar to humans; however, this sweeping generalization cannot be made (58). Despite the lack of evidence, because of receptor and epitope similarity, non-human primates may be an appropriate species for testing certain classes of drugs, for example, large molecule and biological compounds due to the high degree of cross reactivity in those compounds between humans and non-human primates (59).

### Porcine Cancer Models

Swine cancer models are also highly relevant due to their similarity in size, anatomy, pathophysiology, metabolism, genetics, epigenetics, and pathology, as well as their reduced cost compared to non-human primate models (60–68). Swine subjects age at approximately 3–5 times the rate of humans and have similar clinical laboratory and histological findings (66). This life cycle permits enough time to develop, characterize, and modulate cancer in the swine model from weaning to adolescence (69) but also sufficiently short-lived that reasonable research aims and budgets can be outlined and accomplished. Advances in DNA sequencing and our understanding of the role of non-coding DNA sequences have provided insights into the mechanisms underlying altered gene expression and other drivers of cancer development. Swine genetics in particular lends itself to clinically translatable studies due to many available outbred lines. The outbred nature of pigs is key in imitating the variety of genetic profiles underlying human patient populations and cancer types. In addition, the pig genome has high homology with the human genome (70, 71) and epigenetic regulation is highly conserved (67). This elucidation of the porcine genetic profile combined with advances in genetic engineering has permitted the creation of genetically modified pig cancer models that not only follow an analogous disease course as humans (72) but also respond to cancer drug therapy similarly to humans in randomized controlled trials. High-throughput genome sequencing and a collection of precision-genetic tools combined with tools for bioinformatics analyses and profiling of gene expression/proteomics can be applied to pigs (67, 68, 71, 73–77). The ability to modify mammalian genomes through transgenesis, targeted nucleases, and CRISPR, united with the development of advanced reproductive technologies including cloning, allows researchers to create complex and unique cancer models in swine that are more applicable to human malignancies (73, 78). Current porcine models utilized for cancer research include an *APC^1311^* porcine model of familial adenomatous polyposis that produces polyps but not tumors (79), a heterozygous *TP53* knockout model of spontaneous osteosarcomas (80), and a chemically induced porcine HCC model, which takes over 1 year to develop clinically relevant tumors (81, 82).

In addition to recent advances in making precise genetic modifications to pig genomes, there has been significant progress in technologies for testing consequences of genetic changes. Imaging modalities such as computed tomography (CT), magnetic resonance imaging (MRI), and positron emission tomography (PET) can be easily applied to large animals such as pigs, whereas application of analogous clinical protocols is difficult and impractical using rodents (83). By applying these imaging modalities to swine cancer models, detection techniques, progression monitoring, and therapeutic response assessments may be improved. The pig’s size permits radiation-directed therapies to be tested and optimized. Surgical resection is the first line of therapy and often the standard of care for many cancers. The pig’s anatomy allows refinement of surgical techniques and studies of local tumor recurrence both of which are difficult or impossible to perform in rodents. In addition, tumor natural history is an area that is difficult to study in rodents due to their short lifespan, about 1/30th that of humans (7). Swine can live up to 10 years, thereby enabling researchers to carefully follow the development of tumors, tumor progression, invasion, and metastasis in the absence of intervention over time. Additionally, the identification of biomarkers may be more feasible in these animals due to the facile nature of accessing blood and tissue samples, the abundance of sample material and the ability to perform longitudinal blood sampling over longer periods of time. Understanding tumor heterogeneity may be well suited for a large animal, as samples could be collected from many different tumors over time and followed for variations in somatic mutations, gene expression, epigenetic alterations, or differential responses to treatment (66, 78).

One of the main drawbacks of rodent cancer models has been their inability to identify safe and effective drugs to treat cancer. Mouse cancer models have been poor predictors of drug safety, toxicity, and efficacy (84). Furthermore, routes of administration in mice are largely limited to intravenous (i.v.), intraperitoneal (i.p.), or oral gavage. Pigs have been widely used in pre-clinical drug toxicology and are a standard large animal model for pre-clinical toxicology prior to human studies (63). The size and ease in handling pigs allows drugs to be administered in the same manner that patients are administered, including orally, i.v., i.p., by inhalation, dermal absorption, subcutaneous, intramuscular, and transmucosal routes. Longitudinal blood sampling can be performed to assess drug exposure and metabolism over long periods of time, and the amount of blood samples that can be taken from swine in a short period of time enhances the ability of pharmacologists to get precise kinetic data following drug exposure. There are significant homologies between swine and human xenobiotic receptors that regulate drug metabolism and pharmacokinetic properties (85). The cytochrome P450 (CYP) superfamily of proteins plays a critical role in the processing and metabolism of drugs, and again, many studies have shown parallels in the structure and function of these molecules in pigs and humans (85). Importantly, for pediatric cancer drug studies, juvenile pigs have been shown to have similar pharmacokinetic responses to certain drugs that cannot be modeled in other animals (86). Finally, pigs are easily subject to models of relevant comorbidities including non-alcoholic steatohepatitis (NASH) and alcohol-induced cirrhosis. The use of pigs in pre-clinical drug testing may identify safer and more effective therapies as well as establish dosing and routes of administration for new drugs prior to human clinical trials. Practically speaking, enrollment of pigs to clinical research studies eliminates the accrual barrier observed in candidate dogs and human clinical trial patients because cohorts of pigs are accessible. Furthermore, a facile porcine genome engineering platform enables future humanization of drug metabolism in swine models (66).

## The OCM

**Figure 1:**
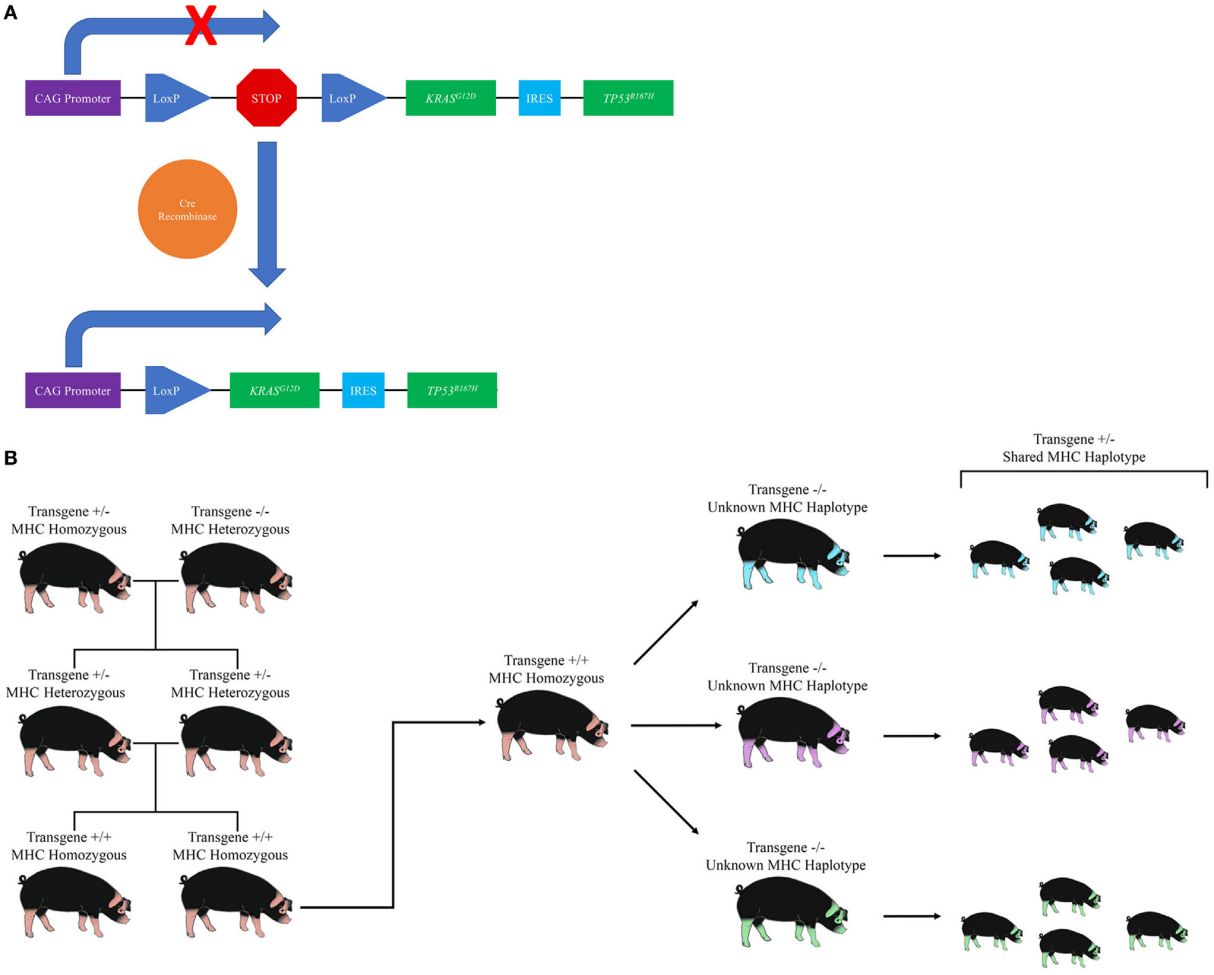
Development and utilization of the Oncopig Cancer Model (OCM). **(A)** Diagram of the Oncopig transgene cassette located on chromosome 18. A Lox-Stop-Lox sequence prevents expression of *KRAS^G12D^* and *TP53^R167H^*. Exposure to Cre recombinase results in site specific recombination between the two recognition sites (LoxP), resulting in removal of the Stop sequence and subsequent expression of *KRAS^G12D^* and *TP53^R167H^*. **(B)** Diagram of the breeding scheme used to produce Oncopigs for experimental use. The original male Oncopig homozygous for an MHC haplotype and carrying a single transgene cassette located on chromosome 18 is bred to a non-transgenic female to produce offspring heterozygous for the transgene cassette and MHC haplotype. The resulting heterozygous offspring are further bred to produce Oncopig offspring homozygous for both the transgene cassette and an MHC haplotype. Male homozygous offspring can then be bred to a variety of transgenic or non-transgenic pig breeds (depicted through varying color) to produce genetically diverse Oncopigs for experimental purposes, all of which harbor a single copy of the transgene cassette and a shared MHC haplotype.

The OCM is a novel transgenic swine model that recapitulates human cancer through development of site and cell specific tumors following Cre recombinase induced expression of heterozygous *KRAS^G12D^* and *TP53^R167H^* transgenes (87). Details regarding the generation of the OCM can be found in Schook et al. (87). Briefly, porcine *KRAS* and *TP53* cDNA were cloned and site-directed mutagenesis was performed to introduce the oncogenic G12D and R167H mutations, respectively. The two cDNAs were then introduced into a Cre-inducible vector containing a CAG promoter followed by a Lox-Stop-Lox (LSL) sequence—which prevents expression of the transgenes until it is removed by Cre recombinase—followed by a single copy of the *KRAS^G12D^* and *TP53^R167H^* transgenes separated by an internal ribosome entry site (IRES) sequence (Figure 1A). Normal Minnesota minipig embryotic fibroblasts were transfected with the resulting plasmid, and stably transfected cells were used as the source of nuclei for somatic cell nuclear transfer (SCNT). A single male Oncopig was selected from the resulting litter to develop the Oncopig herd (Figure 1B) due to the insertion of the transgene construct at a single location on chromosome 18. The breeding scheme depicted in Figure 1B allows the production of a herd of male and female Oncopigs homozygous for both the transgene and an MHC haplotype. The resulting homozygous males can be bred to a wide range of available pig breeds, allowing the production of genetically diverse experimental Oncopigs possessing a single copy of the mutated transgenes, the WT alleles, and a shared MHC haplotype important for immunological studies as described below (Figure 1B).

The *KRAS^G12D^* and *TP53^R167H^* mutations were chosen because the resulting amino acid substitutions are commonly found in human cancers, with *RAS* and *TP53* mutated in one-quarter and one-third of all human cancers, respectively (88, 89). These mutations are also observed simultaneously in human cancers, making this a highly relevant model from a genomics perspective. Utilization of two mutations commonly observed in human tumors allows production of tumors driven by the same molecular alterations as humans in a species with similar anatomy, physiology, metabolism, and genetics. In addition, the heterozygous outbred nature of the OCM means that this model more closely mimics the human condition in comparison to commonly used inbred, homozygous germline mouse models. This model, as a transitional animal model from mice or other small animals to humans, fulfills the currently unmet clinical modeling needs for relevant investigation of both hematologic and solid tumor cancers. With its genetic malleability and predictable behavior, the OCM offers a comprehensive toolset for modeling both human cancers and comorbid disease. Together, this makes the OCM an ideal platform to develop a wide range of cancer models to test new treatments, develop standards, and improve early detection rates. The OCM is a transformational research tool for the investigation of therapeutic efficacy while significantly reducing the costs, variables seen in human subjects, and lengthy conduct of human clinical trials. The following sections discuss progress made to date on modeling various cancer types in the OCM.

### Soft Tissue Sarcomas

Soft tissue sarcomas are a group of rare mesenchymal tumors that carry a 5-year survival rate of 50%. STS consist of over 50 subtypes and arise from a number of tissue types including fat, muscle, blood vessels, and nerves (90). As the survival rate for STS has remained unchanged for decades, there is a critical need for further research into STS characterization and treatment. This research is currently limited by the availability of STS cell lines and tissue samples (91), highlighting the need for transitional STS models for improved STS detection, diagnosis, and treatment. As *TP53* represents one of the most frequently mutated genes in human STS (92, 93), the OCM represents an ideal model to develop STS cell lines and *in vivo* models critical for improving survival rates for patients with STS. To date, both STS cell lines and *in vivo* STS tumors have been developed and characterized in the OCM (76, 87).

Utilization of human tumor cell lines is critical for expanding our understanding of tumor biology and developing new cancer therapies (94). However, given the high number of diverse human STS subtypes and limited cell line availability, additional STS model cell lines are required to investigate the mechanisms underlying variable targeted therapy responses observed across STS subtypes (91, 95). As an initial proof of concept to demonstrate the ability to transform Oncopig mesenchymal cells, fibroblasts isolated from Oncopig skin biopsies were transformed via exposure to Cre recombinase *in vitro* (87). The resulting STS cell lines expressed both *KRAS^G12D^* and *TP53^R167H^* transgenes and displayed tumorigenic phenotypes, including altered morphology, reduced cell cycle length, increased cell migration, and soft agar colony formation (87). Injection of the STS cell lines in SCID mice resulted in tumor formation (87), and transcriptional profiling of Oncopig STS cell lines via RNA-seq identified transcriptional hallmarks of human STS, including altered TP53 signaling, Wnt signaling activation, and evidence of epigenetic reprogramming, including altered expression of DNA and histone methyltransferases (76). In addition, *FOSL1*, a key transcriptional regulator of human STS, was identified as a master regulator in the Oncopig STS cell lines (76), further demonstrating the similarities between Oncopig and human STS at the molecular level. These *in vitro* phenotypes and transcriptional profiles are consistent across replicates and in lines cultured for extended periods of time (76), highlighting the stability of Oncopig cell lines. As the OCM supports the transformation of any cell type, it provides a platform for the production of stable STS cell lines originating from a wide variety of mesenchymal cell types for *in vitro* STS research.

While cell lines are useful for understanding fundamental tumor biology and testing potential new therapies, *in vivo* transitional models are also critical to translate basic *in vitro* discoveries into clinical practice. STS tumor formation has been successfully demonstrated via direct injection of adenoviral vector encoding Cre recombinase (AdCre) into Oncopig skeletal muscle, resulting in tumors blindly pathologically characterized as leiomyosarcomas (76, 87, 96). These tumors develop rapidly and consistently and also recapitulate transcriptional hallmarks of human leiomyosarcomas, including altered TP53 signaling, Wnt signaling activation, and evidence of epigenetic reprogramming (76). Master regulators of Oncopig leiomyosarcomas were also consistent with human leiomysoarcomas, including *MEF2C*, which acts as a tumor suppressor in human leiomyosarcoma (97). The Oncopig leiomyosarcoma model therefore represents a qualified alternative tumor model for pre-clinical treatment and imaging testing, as well as an ideal training tool for surgical and procedural specialties. In fact, the OCM is already being utilized for device testing. Using the Oncopig leiomyosarcoma model, researchers have tested the efficacy of 3D spatially registered real-time image-guided catheter-based ultrasound (CBUS) thermal ablation therapies (96). By inducing leiomyosarcoma formation and then treating these tumors, the ability to utilize 3D tracked ultrasound image guidance to precisely place catheters and treat tumors was demonstrated, resulting in complete ablation of the tumor (96). This demonstrates the ability to utilize the OCM as a pre-clinical model for device testing not possible in small animal models.

### Pancreatic Cancer

Despite modest improvements in recent years, pancreatic cancers remain highly lethal with an overall 5-year survival of 8% (98). Genetically modified mice have allowed tremendous insights into disease etiology, particularly on a genetic level, as well as *in vivo* characterization and mechanisms. The KPC mouse model has been the gold standard for pre-clinical pancreatic cancer research for over a decade. Yet, such models are limited in scope for more direct translational application to humans regarding epigenetic events and therapies given their anatomical and physiological variances compared to humans. Given the greater anatomical and physiological similarities between pigs and humans, an Oncopig pancreatic ductal adenocarcinoma (PDAC) model will provide a more clinically relevant model, allowing insight into surgical and interventional radiology techniques not possible in currently used mouse models. While such a porcine model is certainly not without limitations, the domestic pig may more faithfully recapitulate human PDAC and expand our understanding of disease pathology beyond what is possible using current small animal PDAC models.

A porcine PDAC model is currently being developed using the OCM. Successful induction of both predominant pancreatic cancer histotypes—exocrine and neuroendocrine—via direct delivery of AdCre to the main pancreatic duct has been demonstrated in the OCM. This approach led to locally invasive disease sharing histological hallmarks of human PDAC including a dense fibroblastic stroma and acinar-to-ductal metaplasia (99), which may provide a more clinically relevant model than currently used small animal PDAC models. This is particularly important for the neuroendocrine component, which is relatively underrepresented in research compared to exocrine/ductal cancers. Murine models of PNET produce a variety of PNET types with varying behaviors ranging from indolent to highly aggressive (100, 101). While these models have proved to be valuable prototypes of disease, they are limited in the extent to which they can represent human PNET. The low incidence and heterogeneous presentations of PNET itself combined with the low availability of pre-clinical models have slowed progress in terms of early diagnosis and the development of targeted therapies. In fact, one of the most significant clinical challenges in the management of pancreatic caner is its late presentation, demonstrated by the diagnosis of more than 80% of pancreatic cancer cases at regional and distant stages. As stage is the key prognostic factor in pancreatic cancer survival (98), a means of improved early detection is extremely attractive to clinicians. Given the size and orientation of the pig pancreas, near identical imaging modalities can be used to longitudinally follow disease progression immediately after induction in the Oncopig PDAC model, which may improve our understanding of early events in the carcinogenic process and facilitate earlier detection.

Furthermore, the Oncopig PDAC model also allows investigation into several clinical avenues that are not possible or must be significantly altered to perform in rodents. For instance, patients with locally surgically resectable tumors have improved survival compared to those with inoperable disease (98). However, in rodents, the study of many novel surgical techniques is impossible due to the differences in size and anatomy. In this capacity, the Oncopig PDAC model may allow investigation of new surgical interventions as well as nanotechnology and localized drug delivery methods for pancreatic cancers.

### HCC and Comorbidities

Worldwide, HCC is the fifth most common cancer and the third most common cause of cancer-related deaths, occurring more often in men than in women. In the United States, 40,710 new cancers of the liver and intrahepatic bile duct are expected in 2017, with an estimated 28,920 deaths. HCC is the main form of primary liver cancer that carries a 5-year survival rate of 17.5%. This low survival rate is predominantly due to the low number (15%) of patients who are eligible for surgery or other curative therapies at the time of diagnosis (102), highlighting the need for improved HCC early detection and treatment strategies. A number of locoregional therapies (LRTs) including cryoablation, radiofrequency ablation, and transarterial chemoembolization are currently used to treat HCC patients who are ineligible for surgery; however, the optimum treatment strategy is dictated by the physician’s specialty as opposed to evidence-based consensus (103). This highlights the need for improved HCC animal models to test LRTs and combination therapies to better understand the intrinsic tumor biology underlying differential treatment responses. An Oncopig HCC model would provide an ideal transitional model to address this need as well as refine techniques to help improve early detection rates.

The OCM, as an HCC investigational tool, offers a novel, physiologically and anatomically relevant cancer model for which a multitude of innovative therapeutic modalities can be applied and tested. This model is a critical transitional, translational, and transformational research tool for the investigation of therapeutic efficacy, variables seen in human subjects, and lengthy conduct of human clinical trials. Importantly, it can be utilized to conduct correlative studies for more efficient and consistent investigation of new therapies. Its size allows utilization of the same methods and instruments used in human clinical practice, and the segmental nature of the pig liver (similar to human anatomy) allows each Oncopig to serve as its own therapeutic control. Although the information gained from human clinical studies has been used to marginally enhance the efficacy of current standard of care LRTs, such trials have provided only limited capability for the investigation of the fundamental processes contributing to procedure effectiveness and disease relapse.

An Oncopig HCC model has been initiated to serve as a transitional model linking murine results with clinical outcomes. Oncopig HCC cell lines have been created by isolating hepatocytes from Oncopig livers followed by *in vitro* transformation (77). These cell lines recapitulate human HCC characteristics, including an epithelial-mesenchymal transition, secretion of alpha-fetoprotein (AFP), and transcriptional similarities including *TERT* reactivation, apoptosis evasion, angiogenesis activation, and Wnt signaling activation (77). In addition, direct comparison between Oncopig and 18 commonly used human HCC cell lines revealed conservation of master regulators of gene expression (77). The Oncopig HCC cells also form hypervascular tumors histologically characterized as Edmondson Steiner grade 2 HCC with trabecular patterning when implanted into both SCID mice and Oncopigs subcutaneously (77). In addition, T-lymphocyte infiltration is observed, indicating that these are “hot” tumors potentially appropriate for immunotherapy trials. This is an important aspect of this model, as it is clear that HCC-specific antigens are recognized by the immune system and contemporary clinical studies have indicated that manipulating the immune response can be deleterious to HCC tumor growth (78). Together this suggests that the Oncopig HCC model is a qualified alternative for improving HCC detection, treatment, and biomarker discovery.

In addition to the formation of clinically relevant tumors, an ideal HCC animal model must also mimic relevant comorbidities observed in humans. Alcoholic liver disease and NASH represent common chronic liver ailments, both of which are progressive and incite liver cirrhosis—a precancerous state of liver scarring—that increases the risk for HCC development. A protocol for the induction of alcohol-related liver cirrhosis within 8 weeks using intravascular administration of an ethanol-ethiodized oil emulsion via the hepatic artery has been successfully tested and validated in the OCM (77). This provides the opportunity to assess the role of chronic alcohol-induced liver cirrhosis in HCC tumorigenesis. In addition, researchers have utilized the Ossabaw pig to generate a porcine NASH liver disease model (104). As Ossabaw pigs are genetically predisposed to obesity and diabetes, exposure to a “Western” or “NASH diet” results in the development of severe metabolic syndrome with markedly abnormal liver histology that closely mimics human NASH within 8–24 weeks (104). While this represents a promising porcine NASH model, natural progression to HCC would take years to develop. However, crossbreeding the OCM and Ossabaw would result in a unique “Oncobaw” cross characterized by capacity for inducible tumors as well as development of NASH liver disease, providing a distinctive platform to study HCC in the NASH liver microenvironment.

### Oncopig Immunological Profiling

The hallmarks of cancer have recently been updated to include the ability of cancer cells to avoid immune recognition and subsequent destruction (105). The impact of having immune cell infiltrates at the tumor site has especially been evaluated in colorectal cancer patients, where intratumoral T cells with cytotoxic nature and memory phenotypes allowed prediction of prognosis for patients at an early stage of disease (106). In addition to the *type* of immune cell infiltrates, the outcome for colorectal cancer patients was also found to be dependent on both the *density* and *location* of the immune cells within the tumor (107). Together, these three concepts formed the basis of the *Immunoscore*, which has already become an integrated part of the prognostic approach for colorectal cancers in humans (108). The mechanisms underlying the ability of the cancer cells to avoid immune destruction have been proposed to differ dependent on the degree of immune cell infiltrates (109). For this reason, an immunological characterization of the OCM tumor landscape is considered crucial to subsequently use this information for design of pre-clinical immunotherapeutic studies. Recently, both infiltration of several T-cell subsets within OCM tumors and endogenous anti-tumor immune responses have been demonstrated (Overgaard et al., 2017, submitted). This indicates that the OCM develops “hot” tumors and a porcine version of the *Immunoscore* will indeed be both interesting and useful when selecting which therapies to test in the OCM. Adaptation of an *in vivo* cytotoxicity assay in line with what has previously been demonstrated for mice and monkeys (110–112) would allow a direct measure of anti-tumor cytotoxic immune responses and identification of epitopes involved in the cytotoxic recognition of tumor cells. While T-cell infiltration has been observed in Oncopig tumors developed via either AdCre or transformed cell line injection, comparisons of the resulting tumor heterogeneity and immune microenvironments induced by the two tumor formation methods have not been performed. These future comparisons will be important for ensuring Oncopig tumor heterogeneity, the surrounding microenvironment, and subsequent immunological responses mimic those observed in humans.

In addition, adoptive transfer of T cells between MHC-matched Oncopig littermates is now a possibility with the development of a NGS-based approach for porcine MHC class I allele typing (113). Those animal pairs suddenly enable adoptive T-cell therapies to be tested in a large and fully immunocompetent animal model. Finally, in addition to the adaptive immune system, the porcine innate immune system has been heavily studied and found to be similar to humans in terms of anatomy, organization, and response (114). For example, pattern recognition receptors such as toll-like receptors (TLRs) have been heavily studied in pigs (115–117), providing insights into their evolution, variability across breeds, and similarities with humans. In addition, vast knowledge regarding porcine cellular and humoral innate immune responses and their similarities with humans exist (114). While this work has not been performed in the Oncopig, the extensive knowledge of both the porcine adaptive and innate immune system represents a significant advantage for this and other porcine cancer models, given the emerging role of the immune system in tumor development and treatment.

## Utilizing the OCM to Address Unmet Clinical Needs

Animal research has played a vital role in advancing biomedical science. However, laboratory animals may experience significant adverse effects as a result of experimentally induced cancers and the effects of investigative or treatment regimes are substantial (118, 119). Therefore, the use of animals in research comes with ethical responsibilities (120). The three R’s (Reduce, Replace, and Refine) as defined by Russell and Burch (121) provide a practical strategy for applying an ethical framework to animal research. These guiding principles indicate that researchers must seek to (1) replace animal use with alternative techniques, (2) reduce the number of animals used to the minimum required to obtain meaning information, and (3) refine experimental procedures to ensure animal suffering is reduced as much as possible.

Consistent with the three Rs, the Oncopig model allows the discrete induction of localized tumors that can be closely followed to meet scientific objectives while minimizing comorbidities and mortality. However, in order for the OCM to be utilized to its full potential, an understanding of how the various Oncopig-based cancer models can be applied to specific unmet clinical and pre-clinical human cancer needs is required. This section describes significant and pressing unmet clinical needs that (1) need to be addressed to improve disease burden and survival rate and (2) can be effectively addressed by utilizing the OCM as a translational model to bridge the gap between small animal models and human clinical trials.

### Early Detection

**Figure 2:**
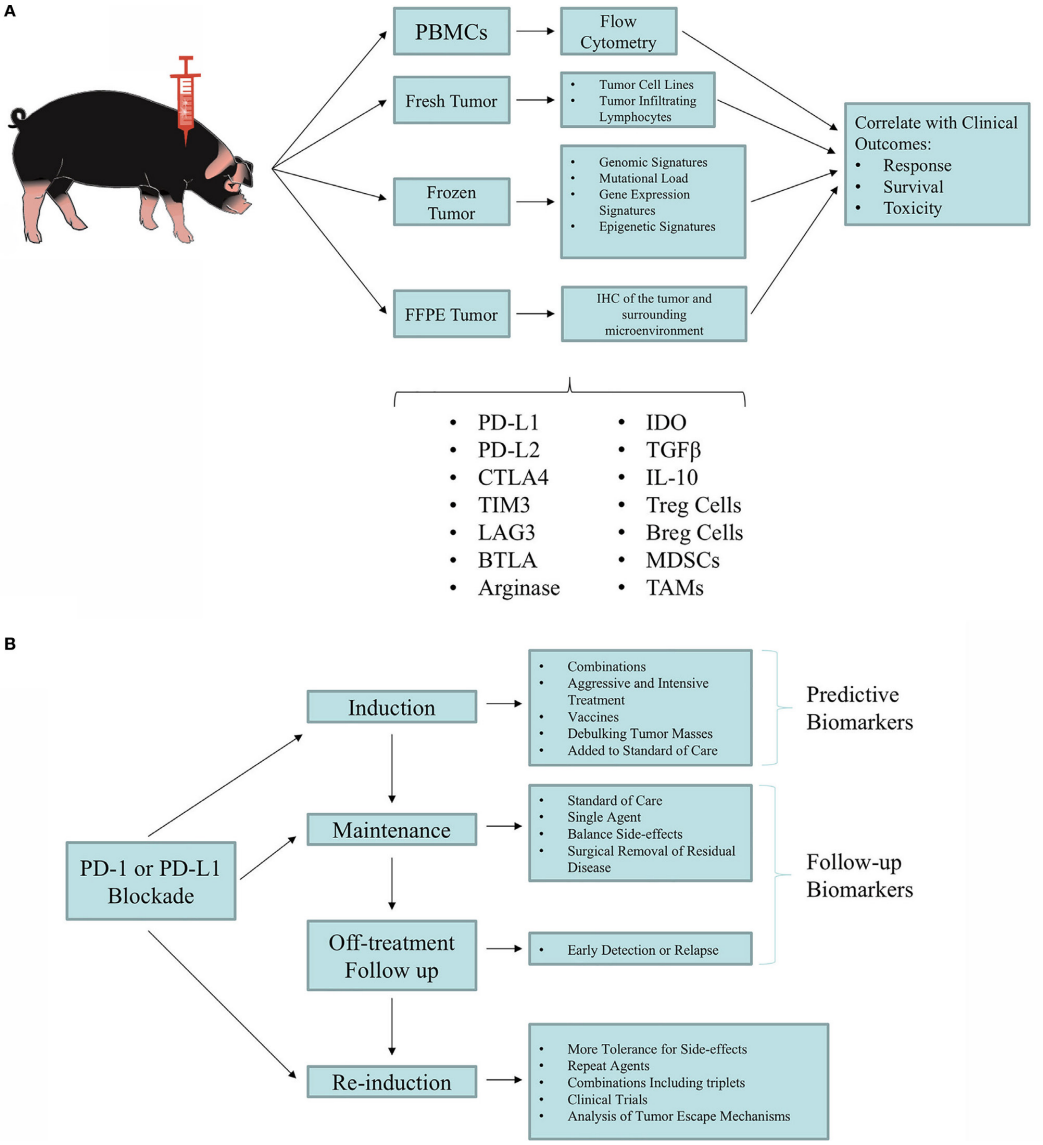
Utilization of the Oncopig Cancer Model (OCM) for biomarker discovery and validation. **(A)** Tumor and blood samples are taken from Oncopigs for biomarker screening studies. Samples are processed immediately (fresh), flash-frozen, or formalin-fixed paraffin-embedded (FFPE). Blood samples can immediately undergo Ficoll density centrifugation for isolation of peripheral blood mononuclear cells (PBMCs). Fresh tumor samples can be used to produce tumor cell lines and isolate tumor-infiltrating lymphocytes (TILs). The flash-frozen samples can be used for deep sequencing (i.e., whole-exome sequencing, RNA-seq, miRNA-seq, and DNA methylation analysis). FFPE samples can be utilized for immunohistochemistry (IHC) of the tumor and the tumor microenvironment. Results obtained from these analyses can be correlated with patient outcomes to identify predictive biomarkers. **(B)** Proposed testing of clinical management with immunotherapy. As new immunotherapy agents and combinations are developed, optimal combinations and subtype susceptibility must be determined. Patients experiencing durable responses that are sustained even off treatment require new concepts in risk management and mitigation, while making the most of the clinical benefit. Overall, a phased approach can be tested in which aggressive combination regimens that achieve frequent responses can be followed by maintenance with less aggressive and safer regimens, reaching the point of weaning responsive animals off treatment. Identifying biomarkers will be crucial for optimal clinical management. Adapted from Ref. (122).

As the biomedical research community investigates the diagnostic and prognostic value of liquid biopsies, a large animal model becomes increasingly important for both metabolic similarities and ease of sampling. Candidate biomarkers from serum, plasma, or peripheral blood must be accurately and reproducibly measurable, clinically feasible, cost-effective, and prospectively validated in randomized clinical trials. Due to the inducible nature of the OCM, this model represents an ideal large animal model for the identification of candidate early detection biomarkers. Figure 2 outlines a variety of biomarkers that have been validated in the clinic and can be more rigorously tested in the OCM. Putative biomarkers in blood consist of soluble factors such as serum proteins and circulating tumor DNA, or other cellular factors such as tumor cells, T-cell subsets, and other immune cell populations. The serum factors may be single or could include a panel of factors preferably measured by a single, validated assay. To date, most published analyses of peripheral blood biomarkers in immunotherapy have been retrospective and hypothesis generating, although important information has been gained that illuminates the mechanisms of clinical benefit with some approaches and has helped inform subsequent clinical trial design.

### Immunogenicity and Immunotherapy

After decades of research, the hope of effective immunotherapy for solid tumors became a reality with the development of immune checkpoint inhibitors (78, 123). This elegant approach leverages the immune system, which has the capability to recognize a diverse array of both foreign and tumor-derived antigens, to exact a tumor-specific response capable of arresting malignant growth. Novel immunotherapeutic regimens that both counteract these immunosuppressive mechanisms and amplify tumor-specific immunity have the potential to profoundly improve clinical outcomes for cancer patients. The recent demonstration that cancer immunotherapy extends patient survival has reinvigorated interest in elucidating the role of immunity in tumor pathogenesis. Since ipilimumab entered the treatment landscape in 2011, immunotherapy has continued to revolutionize cancer therapy. In fact, immunotherapy was named the American Society of Clinical Oncology (ASCO) top cancer advance of the year for 2016 (124). A number of U.S. FDA-approved agents have become available for an increasing number of difficult-to-treat cancers, such as melanoma, renal cell carcinoma (RCC), HCC, and lung cancer, among others. In contrast with most chemotherapy and targeted therapies, immunotherapy offers the possibility of durable responses, sometimes even without continued treatment (125–127). However, objective responses among patients treated with single-agent regimens are seen in less than one-half of patients treated. Combination of immune checkpoint inhibitor therapy raises response rates but also increases toxicity and cost (128). Thus, to optimize selection of appropriate patients for immunotherapy and avoid unnecessary toxicity and health care costs, there is a clear need to identify truly predictive, and not simply prognostic, biomarkers of response. The OCM can address some of the issues regarding selection of agents and expected immune responsiveness as novel agents are developed.

Understanding which factors predict clinical benefit with immunotherapy in a relevant animal model can improve the selection of tumor types and patient subsets who will respond, illuminate the mechanism of action of novel immunotherapeutic approaches, and potentially inform which patients require single-agent versus combination strategies (Table 1). Examples of biomarkers in the immunotherapy landscape include (1) soluble factors such as serum proteins, (2) tumor-specific factors such as receptor expression patterns and components of the microenvironment, (3) identification of immune cell subsets such as Treg, and (4) host genomic factors (129–131). Despite the interest in biomarker development for immunotherapy, validated biomarkers have remained an elusive goal and the availability of the OCM enables biomarker validation of serum, immune cell, tumor, and tumor microenvironment to be correlated with response.

**Table 1:** Potential predictive biomarkers for immunotherapy.

Type	Source	Biomarker	Clinical significance
Liquid	Serum	IL-6	High-dose IL-2 treatment failure and shorter overall survival associated with high levels in metastatic renal cell carcinoma
		CRP	High-dose IL-214 resistance associated with high levels; decreasing levels during ipilimumab therapy associated with disease control and survival
		VEGF	Lack of response to high-dose IL-2 is associated with high levels and decreased overall survival
		LDH	Ipilimumab therapeutic benefit predicted by low pretreatment levels; decreasing levels during ipilimumab therapy associated with disease control and survival
		sCD25	Ipilimumab therapy resistance predicted by high levels
		NY-SEO-1 antibody	Greater likelihood to respond to CTLA-4 blockade predicted by seropositivity

Cellular	Peripheral blood	Neutrophils/leukocytes	High-dose IL-2 treatment failure and shorter overall survival associated with high counts
		Lymphocytes	High-dose IL-2 therapy response associated with immediate lymphocytosis
		CD8+ T cells	Clinical benefit to CTLA-4 blockade associated with presence
		Absolute lymphocyte count	Increasing counts during ipilimumab therapy associated with improved overall survival
		Eosinophils	Increasing counts during ipilimumab therapy associated with improved overall survival
		CD4 + ICOS + T cells	Increase in frequency after ipilimumab therapy
		Myeloid-derived suppressor cells	Ipilimumab therapy benefit predicted by low frequency
	Tumor	PD-L1	
	Tumor-infiltrating lymphocytes	CD4 + ICOShigh T cells	Clinical benefit of ipilimumab correlated with increased frequency
		CD8 + T cells	PD-1/PD-L1 expression predicts response to PD-1 blockade

Genomic	Tumor	Tumor mutation loads	Predicts clinical benefit of ipilimumab and PD-1 blockade
		Mismatch repair	Predicts clinical benefit of PD-1 blockade

*Adapted from Ref. (132)*.

Our incomplete understanding of the mechanisms of action of specific immunotherapies makes it difficult to identify a surrogate marker that adequately captures the process across different classes of drugs (133). Many published analyses of potential predictive biomarkers for immunotherapy are retrospective, with limited extension into large prospective trials. In addition, there has been substantial variability in standardization, measurement, and interpretation of early biomarker assays (134). Furthermore, biomarker development in immunotherapy is challenged by the fact that immunotherapy targets are often inducible and dynamic over time and location. This is a function of the complex tumor microenvironment and the contribution of immuno-editing to the immune milieu. The tumor microenvironment involves complicated interactions between several types of infiltrating immune cells such as monocytes, neutrophils, dendritic cells, T and B cells, eosinophils, basophils, mast cells, and natural killer cells, as well as the heterogeneous tumor cells themselves and their companion stromal cells, including tumor-associated macrophages, fibroblasts, adipocytes, endothelial cells, and others (135). The local environment is further complicated by “micro-niches” created by alterations in perfusion, oxygenation, electrolyte levels, and the subsequent development of resistant tumor cells surviving in nutrient- and oxygen-deprived conditions (135, 136). Thus, these micro-niches likely represent distinct microenvironments with different cell types and factors, all within one tumor deposit. Finally, incomplete immune editing may result in selective pressure on tumor cells, resulting in resistant tumor cell clones and immune escape (132, 135).

Despite these challenges, clinical research of immunotherapy over the last several years has confirmed the importance of tumor-infiltrating lymphocytes as both prognostic and predictive indicators for patients with cancer and for treatment with immunotherapy. There have also been several trials of T-cell checkpoint inhibitors in which PD-L1 expression in the tumor microenvironment has been associated with more favorable outcomes, although this has not been uniformly demonstrated. Other groups have used a larger panel of gene signatures, including Treg, CD8, cytokines, chemokines, and other factors, that correlate with therapeutic responses. These studies collectively suggest that there may be host, tumor, and immune factors that can be used for biomarker development. The importance of the tumor microenvironment has been appropriately stressed, but, practically, the ability to use serum or peripheral blood biomarkers is challenging and would be bolstered through utilization of the OCM.

### Therapeutic Screening and Development

During early drug development, the primary goal of testing is to determine if the compound exhibits pharmacological activity that justifies commercial development. If so, the drug then moves into testing for safety. Minimum requirements for drug toxicity testing in non-clinical studies are regulated by agencies such as the FDA in the USA, the Committee for Medicinal Products for Humane Use in Europe, and the ministry of Health, Labor and Welfare in Japan. Common expectations for these agencies are provided by the International Committee on Harmonization (ICH^2^), which has developed standards for acceptable practices in drug development. ICH requires toxicity testing in two relevant animal species (137). Relevant animal species usually include one rodent, either a rat or mouse, and one large animal species. However, many of the available pre-clinical animal cancer models offer limited benefit for therapeutic screening, dosing, and development due to their lack of similar size and drug metabolism compared to humans.

Several factors are considered when selecting the large animal species. Ethical and legal considerations encourage use of the lowest sentient species that will accomplish the scientific goals (121). Other criteria used in selecting a species include the generation of a similar metabolic profile to humans, appropriateness of the species for use in the laboratory environment, prior history of the species with similar classes of drugs, historical database, genetic and phenotypic variability of the species, and/or breed, ease of handling, source, and supply. Swine are not typically used during early drug development because of their large mass and the relative lack of drug until production scales up (138). However, swine have been used in safety assessment and are increasingly used because of similarities to humans in cardiovascular anatomy and physiology, integumentary system, digestive system, renal system, and immune system (139). The porcine *PXR* gene regulates hepatic genes involved in metabolism and transport. *PXR* activates *CYP3A*, which is involved in more than 50% of xenobiotic metabolism, by binding to its regulatory region. The porcine *PXR* gene is 87% homologous to human *PXR*, which represents a significant advantage compared to the 77% homology observed between human *PXR* and mouse *Pxr*. The OCM therefore represents an ideal model for the investigation of absorption, distribution, metabolism, excretion, and toxicity. In addition, the similar size between the OCM and humans, in contrast to small animal models, allows more accurate testing of optimal dose in a pre-clinical setting. Finally, as cancers in humans develop over many years on the background of comorbid disease, utilization of the OCM enables therapeutic screening and development in a setting closely mimicking molecular and clinical backgrounds. Therefore, the OCM represents an ideal model for the investigation of drug metabolism and toxicity predictive of human outcomes.

### Prognostic Indicators

In an ever-changing world of medical research and disease treatment, prognostic indicators evolve as treatment avenues evolve. One key unmet clinical need that biomedical models can bridge is the investigation of prognostic indicators, thereby allowing clinicians to more accurately forecast patients’ benefits resulting from treatment. As mentioned earlier, the OCM allows ample biospecimen (blood, saliva, urine, tissue, etc.) sampling and analysis. Furthermore, given the outbred nature of this model and the adequate supply, the OCM allows cohort investigation, which can provide sufficient unique analysis and volume to determine such prognostic indicators as progression-free survival (PFS) and time to progression (TTP). Based on the incidence of comorbid diseases, such as alcoholic cirrhosis and NASH, the OCM will allow precise diagnosis and prognostication.

### Improved Imaging

The importance of diagnostic imaging in both research and treatment cannot be overstated. Medical imaging, in clinical practice, is used to diagnose, assess, and prognosticate patients’ health over time. The size and anatomy of the OCM provides the ability to easily image—with various modalities including CT, MRI, PET, and ultrasound—in a similar setting as clinical practice. As a model of disease, OCM imaging can be correlated with physical dissection to validate imaging findings, which cannot be done in human patients. Furthermore, the long lifespan and relatively low cost of the OCM permits frequent imaging and longitudinal studies from premalignant through metastatic disease states, which can assist in prognostic assessment and therapeutic response evaluation. The similar anatomy of the OCM allows deep investigation of angiographic imaging, which is limited or impossible in small animal models but is important for inducing disease as well as therapeutic research, as discussed in the following section.

### Device Testing and Surgical Practice

Advancements in surgical technologies are necessary to improve outcomes for patients; however, pre-clinical models in which to test new strategies are limited. One aspect of testing new techniques or devices is the engineering capabilities or technical feasibility of the instrument or procedure; however, equally as important are the ergonomics and ability to translate the findings to human patients. Thus, selecting the appropriate animal model is one of the most important components of pre-clinical testing for these indications, and the animal model chosen should reflect the target patient population.

For a surgical device, technique, simulation, or practice, it is imperative that the anatomy, physiology, and disease state of the animal be similar to humans. As the size of medical devices and instruments used are optimized for human sized organs, a large animal model is ideal within ethical, safety, and financial considerations. Given the similarity of size of the swine organs, skin characteristics, and physiology/immunity; porcine models have become standard in multiple settings including cardiac/atherosclerosis (140), hernia, foregut, transplant (141), hepatobiliary (142), and minimally invasive surgery training and research programs (143, 144). As pigs are true ominvores, the physiology of digestion and liver metabolism are quite homologous, thus rendering the pig a valuable model for translational surgical research. However, what has been missing up until this time is a reliable model in which cancer treatment and resection could be tested. Though clearly this is valuable for an anti-tumor systemic treatment model, it is invaluable for the surgeon who wishes to test a catheter-based, resection-based, or technical procedure since the same instruments used in the human can be utilized in the animal. Furthermore, the OCM allows realistic tumor modeling in which tissue characteristics as a result of tumor growth are reliably recreated and margins can be assessed, both of which have been difficult to model by either orthotopic injections or biomaterial injections (145).

The OCM platform has already been applied to establish STS, HCC, and PDAC in target organs and, using the same sequence of gene mutations, is being used to create colon and other cancer models. Open, laparoscopic, and robotic liver, stomach, pancreas, small bowel, colon, and gallbladder resections have been performed on the OCM using the same instruments, devices, and techniques used in humans, including vascular staplers and energy devices. The size of sutures used in the OCM is the same as humans, and tissue characteristic are near identical from a surgical perspective. Therefore, we see the OCM as an ideal model for surgical technique and device testing in the management of cancers in multiple organs, recapitulating the human situation and allowing realistic and accurate practice in an animal model.

### Development of Standards

The importance of standards development in the field of medical research is understood by veterinary and human clinical researchers alike. The National Cancer Institute supports standards’ development throughout the cancer continuum, including such initiatives as the Veterinary Cooperative Oncology Group (VCOG), the cancer Data Standards Registry and Repository (caDSR), the National Clinical Trials Network (NCTN), and the Genomic Data Commons (GDC), to name a few. Standards, which can refer to data elements, data types and formats, programmatic interfaces, and operating procedures, enable potential data sharing across institutions, diseases, and research studies. With this in mind, the OCM platform was designed to develop and adhere to standards from the beginning. The OCM is part of a centralized platform that includes participating in clinical laboratory assessments, central data submission and management, a central imaging repository, and shared standard operating procedures. With the development and implementation of such standards, any present or future collaborator on OCM projects will have an additional dimension of comparative assessment for study validation.

## Future Modeling Capabilities

In addition to the work currently underway to utilize the OCM to model the abovementioned cancer types, opportunity exists to utilize the OCM to model a wide range of additional cancers. While successful *in vitro* transformation and *in vivo* tumor formation has already been demonstrated in the OCM for three cancer types (STS, PDAC, and HCC), the ability to induce tumorigenesis in any cell type in a temporal and spatial manner provides the framework for modeling cancer types of all origins. While an exhaustive attempt to isolate and transform all OCM cell types has not been performed, to date researchers have not encountered an OCM cell isolate that has not been rendered tumorigenic following exposure to Cre recombinase (unpublished data; Table 2). The ability to transform all OCM cell types attempted to date highlights the potential for utilization in studies focused on additional cancer types, including colorectal, ovarian, fallopian tube, renal, bladder, and skin cancers.

**Table 2:** Oncopig cell isolates successfully transformed *in vitro*.

Cell type/origin	Isolated	Transformed
Fibroblasts	Yes	Yes
Hepatocytes	Yes	Yes
Pancreatic ductal cells	Yes	Yes
Dermal epithelial cells	Yes	Yes
Splenocytes	Yes	Yes
Ovarian surface epithelial cells	Yes	Yes
Fallopian tube secretory epithelial cells	Yes	Yes
Renal proximal tubule epithelial cells	Yes	Yes
Bone marrow (no specific cell isolation)	Yes	Yes
Testis (no specific cell isolation)	Yes	Yes
Skeletal muscle (no specific cell isolation)	Yes	Yes

*List of OCM cell types for which isolation and transformation have been attempted*.

In addition, the OCM is emerging as an excellent candidate for modeling leukemia, lymphoma, and other hematological cancers and their clinically associated comorbidities including obesity, myelodysplasia, age-related changes, and toxin-induced malignancies. Hematological malignancies in swine—reviewed in Ref. (146)—were first reported as early as 1865 (147) but as of yet, there is no porcine model of hematological malignancies that can be reliably induced and consistently reproduced. There is a wide spectrum of potential immunotherapy targets, cellular therapies, and gene targets that can be used to eradicate or control malignant hematopoietic stem cells. However, these therapies present significant safety challenges for patients that cannot be addressed by traditional procedures and require the development of new biomarker protocols and test systems, for which the rigorous use of large animal species will be required. A significant hindrance to development of therapies for hematological malignancies is the limited ability to detect, monitor, and quantify the etiology of hematological malignancies *in vivo*. Indeed, while current imaging strategies increase the predictive accuracy of new drug candidates, they are unsuitable for evaluating minimal residual disease, the foremost problem in current AML therapy. Temporal imaging of the OCM over the course of a disease or treatment regime would allow researchers a better appreciation of disease pathology, response to treatment, and drug pharmacokinetics. In addition, the OCM permits access to blood and bone marrow components, lymph nodes, spleen, and thymic tissue, allowing transformation of these multiple sources of hematopoietic cells. Moreover, the recent development of a porcine CD34 monoclonal antibody (Ozer et al., 2017, submitted) as well as the cloning of additional porcine hematopoietic cytokines and growth factors will enable studies of the regulatory aspects of leukemia and lymphoma development.

The ability to model these and other cancer types in the OCM is further facilitated by the ability to cross the OCM with other breeds, such as the Ossabaw, as well as other transgenic porcine models like the *APC^1311^* porcine model of familial adenomatous polyposis (79). In addition, the successful utilization of CRISPR technology in pigs provides the opportunity to add additional mutations to the OCM background, allowing modeling of the same cancer type with varying underlying driver mutations, as well as cancer types with known genetic backgrounds. Finally, utilization of nanoparticle delivery systems can be utilized to selectively target Cre exposure *in vivo* to specific organs and cell types (148), allowing autochthonous tumor formation of known cellular origin. Together, this highlights the current and future capabilities of the highly customizable OCM to drive transitional cancer research and address unmet clinical needs.

## Author Contributions

KS, RS, JN, NK, MR, NM-E, PG, DP, AP, NO, GJ, KG, AM, LR, HO, RG, and LS wrote and approved the manuscript.

## Conflict of Interest Statement

The authors declare that the research was conducted in the absence of any commercial or financial relationships that could be construed as a potential conflict of interest.

## Footnotes

^1^https://ccr.cancer.gov/Comparative-Oncology-Program.

^2^http://www.ich.org/home.html.
